# Dimensional variability of orthodontic slots and archwires: an analysis of torque expression and clinical implications

**DOI:** 10.1186/s40510-020-00333-5

**Published:** 2020-09-14

**Authors:** Michele Tepedino, Giordano Paiella, Maciej Iancu Potrubacz, Annalisa Monaco, Roberto Gatto, Claudio Chimenti

**Affiliations:** 1 Department of Biotechnological and Applied Clinical Sciences, University of L’Aquila, Viale S. Salvatore, Edificio Delta 6, 67100 L’Aquila, Italy; 2 Department of Life, Health and Environmental Sciences, University of L’Aquila, Piazzale Salvatore Tommasi 1, 67100 L’Aquila, Italy

**Keywords:** Dimensional variability, Third-order clearance, Torsional play, Real torque expression

## Abstract

**Background:**

The loss of third-order information in pre-adjusted brackets due to torsional play is a problem in clinical orthodontics. The aim of this study was to evaluate the impact of slot height, archwire height, width and edge bevel’s radius on the torsional play for three brackets/archwire systems.

**Methods:**

Ninety brackets with a 0.022 × 0.028 in. slot with McLaughlin-Bennett-Trevisi prescription from three different manufacturers were selected, and the slot’s height and depth were measured using a profile projector. Sixty stainless-steel rectangular archwires from three different manufacturers were sectioned and observed with a SEM to measure their height, width, and radius of edge bevel. The recorded data were used to calculate the theoretical torsional play between different slot−archwire combinations. One-way ANOVA was used to compare the measurements within different bracket types and among different manufacturers.

**Results:**

Slot height was usually oversized. Archwire’s height was usually undersized, but oversized wires were also observed. The radius edge bevel was the most variable parameter. A certain degree of torsional play is always present that differs from one bracket type to another of the same producer and that can even be doubled from one manufacturer to another.

**Conclusions:**

Due to production tolerance, differences between the nominal values and the real dimensions of any components of a slot/archwire system are common. This results in a torsional play that limits torque expression. The archwire’s edge bevel plays an important role in torque expression, and clearer information should be provided by the manufacturers regarding this aspect.

## Background

The key factors for a successful outcome of an orthodontic treatment are a careful diagnosis, the patient’s compliance, an accurate treatment planning, and the coherent application of an adequate biomechanics. When using straight-wire appliances, a satisfying outcome depends, among other things, on a precise expression of the bracket’s prescription, which is a result of the bracket positioning, the mechanical properties of the archwire, and the precision of the slot [1]. In particular, clinicians are always struggling to achieve the full expression of the bracket’s third-order information, which is crucial to obtain a correct torque of the anterior and posterior dentition and is highly dependent on the archwire’s alloy properties [2] and a tight slot/archwire coupling [1–3].

This intimate fit is seldom achieved because there is always a variable lack of contact between the bracket’s slot and the archwire that limits tooth movement control. In this situation, to completely express a torque’s prescription, the wire must be twisted with a deviation angle, which is called torsional play [4, 5].

Large torsional play values result in an ineffective or in a slow orthodontic treatment [6], because the clinically achieved torque will be equal to the bracket’s torque minus the amount of torsional play.

The presence of this torsional play depends on both the ligation method and the accurate respect of the nominal dimensional values of slots and archwires [7].

Some modification of the appliance’s size, morphology, or surface finish are linked with the manufacturing process: moulding, for example, is associated with expansion and shrinkage, while milling can produce absorption of grains resulting in a rough surface [2]. There are some technical standards, like the ISO standards (International Organization for Standardization) that regulate the dimensional parameters and the tolerance limits that every industrial product must respect: in the orthodontic field, there are the ISO 15841 (https://www.iso.org/standard/62223.html) for archwires and the ISO 27020 (https://www.iso.org/standard/72549.html) for brackets and tubes.

There is agreement in the literature that real and nominal dimensional parameters do not match because manufacturers do not always respect tolerance limits or because these limits are too broad [1, 3, 4, 6, 8, 9].

While other factors are under the clinician’s control, such as the ligation method, the dimensional accuracy of the appliance is an independent factor that can complicate the clinician’s work, considering how torque control is important to achieve a good occlusion [10, 11], and that several orthodontic mechanics (i.e. intermaxillary elastics, powerchains, and many others) produce a loss of torque, which should be counteracted by the bracket’s torque or the archwire’s incorporated torque.

To our knowledge, the previous existing literature investigated the torsional play of different archwires or brackets, but the effective combination of archwire and slot dimensional variability in a single manufacturer’s system, comprehending also the edge bevel’s radius, has never been evaluated.

The aim of the present study was therefore to evaluate the dimensional variability of pre-adjusted brackets and 0.019 × 0.025″ and 0.021 × 0.025″ archwires from three manufacturers, and the consequent theoretical torsional play for each system.

## Methods

**Fig. 1:**
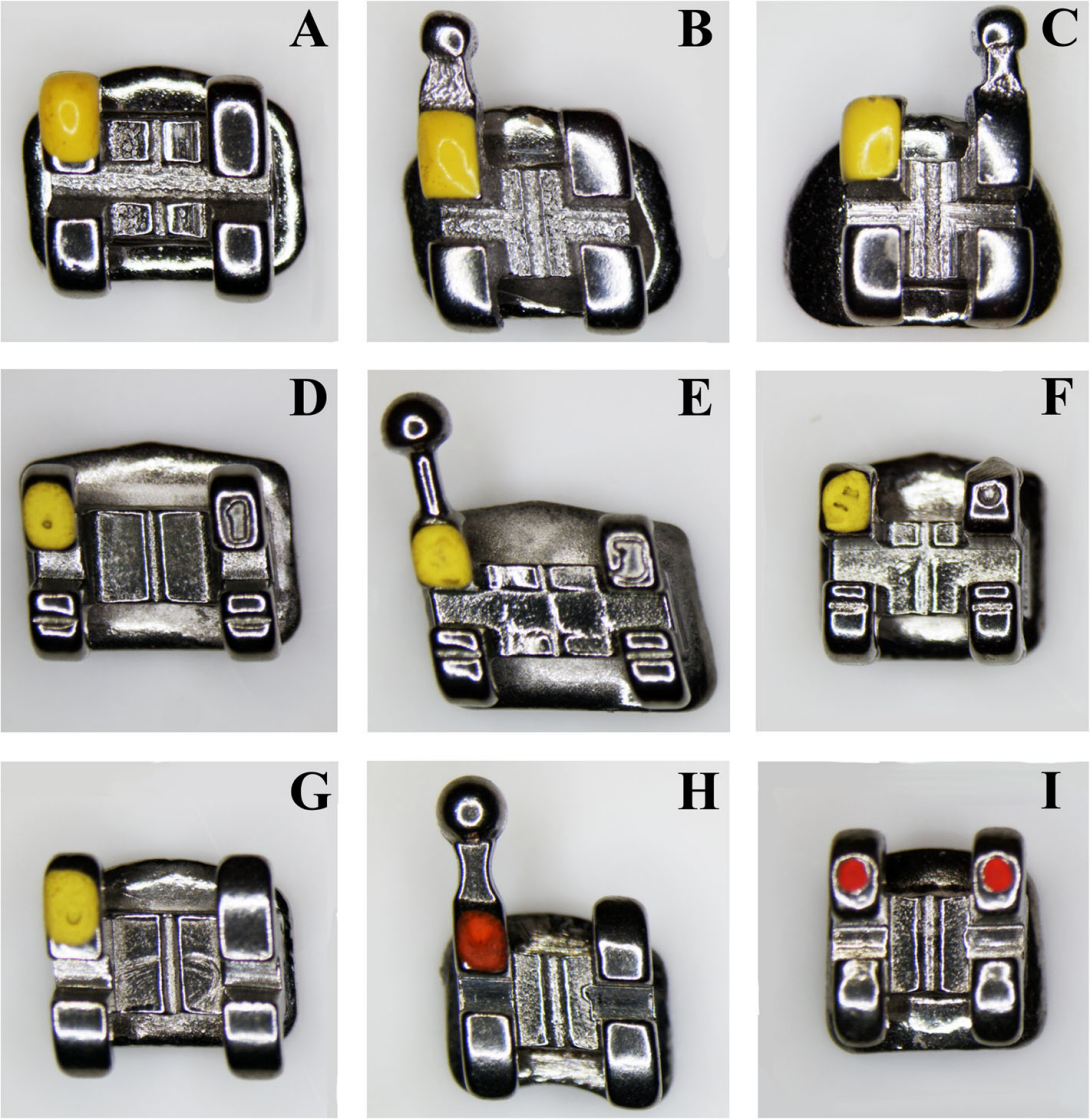
The brackets included in the present investigation. **a** Upper right central incisor from Group 1 (Astar Orthodontics Inc., Shanghai, China). **b** Upper right canine from Group 1. **c** Upper right first premolar from Group 1. **d** upper right central incisor from Group 2 (Sia Orthodontic Manufacturer S.r.l., Rocca D’Evandro, Caserta, Italy). **e** Upper right canine from Group 2. **f** Upper right first premolar from Group 2. **g** Upper right central incisor from Group 3 (Sweden & Martina S.p.A, Due Carrare, Padova, Italy). **h** Upper right canine from Group 3. **i** Upper right first premolar from Group 3

Sample size calculation (G*Power version 3.1.9.2, Franz Faul, Universität Kiel, Germany) [12] revealed that to detect an effect size *f* of 0.916—determined from pilot measurements—with an α probability of 0.05 and a power of 0.95, a total of 24 observations would be needed; therefore, a sample size of 10 specimina per group was considered adequate.A sample of 90 brackets and 60 archwires from three different orthodontic manufacturers was studied: every manufacturer provided 30 brackets (ten for the upper right central incisor, UR1; ten for the upper right canine, UR3; ten for the upper right first premolar, UR4) and 20 rectangular stainless steel archwires (ten 0.019 × 0.025″ archwires, and ten 0.021 × 0.025″ archwires). One operator (CC) coded with numbers all the specimens to mask brand and commercial names, to ensure the blinding of all the other operators. The brackets and the archwires from manufacturer 1 (Astar Orthodontics Inc., Shanghai, China) were coded as Group 1; the brackets and the archwires from manufacturer 2 (Sia Orthodontic Manufacturer S.r.l., Rocca D’Evandro, Caserta, Italy) were coded as Group 2; and the brackets and the archwires from manufacturer 3 (Sweden & Martina S.p.A, Due Carrare, Padova, Italy) were coded as Group 3 (Fig. 1).

The brackets from Group 1 had a 0.022 × 0.028″ slot with McLaughlin-Bennett-Trevisi (MBT) prescription, with torque in base, and were produced by machine milling (Thino™ Low Profile, Astar Orthodontics Inc. Shanghai, China); the brackets from Group 2 had a 0.022 × 0.028″ slot with MBT prescription, with torque in base, and were produced by metal injection moulding (MIM) with a computer numerical control (CNC) milled slot (Supertech bracket, Sia Orthodontic Manufacturer S.r.l., Rocca D’Evandro, Caserta, Italy); the brackets from Group 3 had a 0.022 × 0.028″ slot with MBT prescription, with torque in base, and were produced by the MIM process (PRIMO bracket, Sweden & Martina S.p.A, Due Carrare, Padova, Italy).

Different lots of brackets and archwires had been requested to account also for an inter-lot variability [13], but this was not always possible due to technical reasons, since sometimes each lot is so large that it is difficult to retrieve specimens from a large number of different lots. Lot variation is reported in Table 1.

**Table 1:** Lot variations of the included specimina

	*Group 1*	*Group 2*	*Group 3*
UR1	1	1	3
UR3	1	1	2
UR4	1	1	4
0.019 × 0.025 Archwire	1	1	3
0.021 × 0.025 Archwire	1	1	1

Lot numbers of each sample for every manufacturer

*Group 1* Astar Orthodontics, *Group 2* SIA, *Group 3* Sweden & Martina, *UR1* upper right central incisor bracket, *UR3* upper right canine bracket, *UR4* upper right first premolar bracket

### Slot measurements

**Fig. 2:**
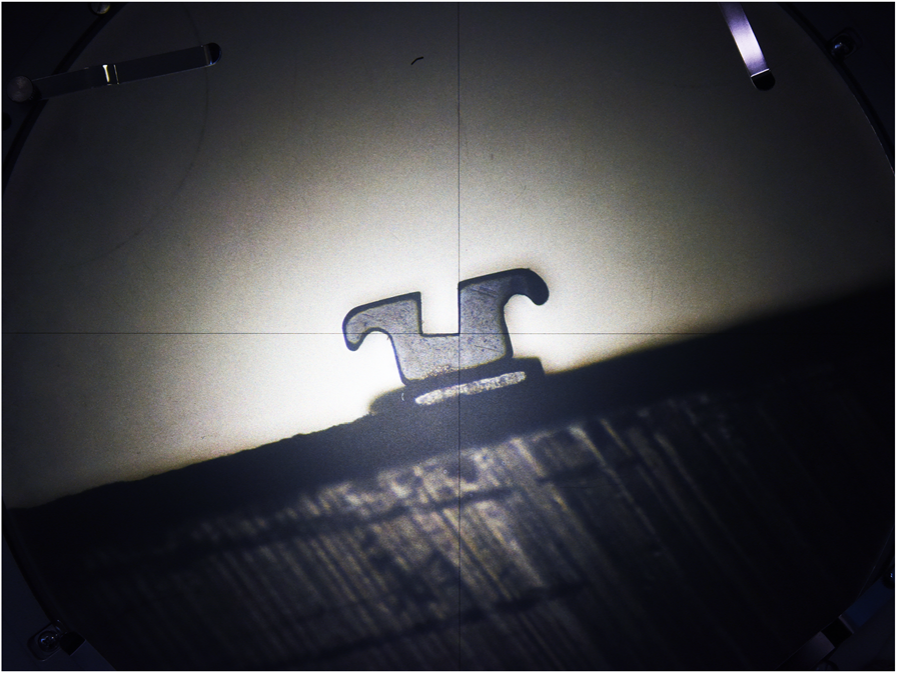
Measurements of the brackets’ slot with a profile projector

**Fig. 3:**
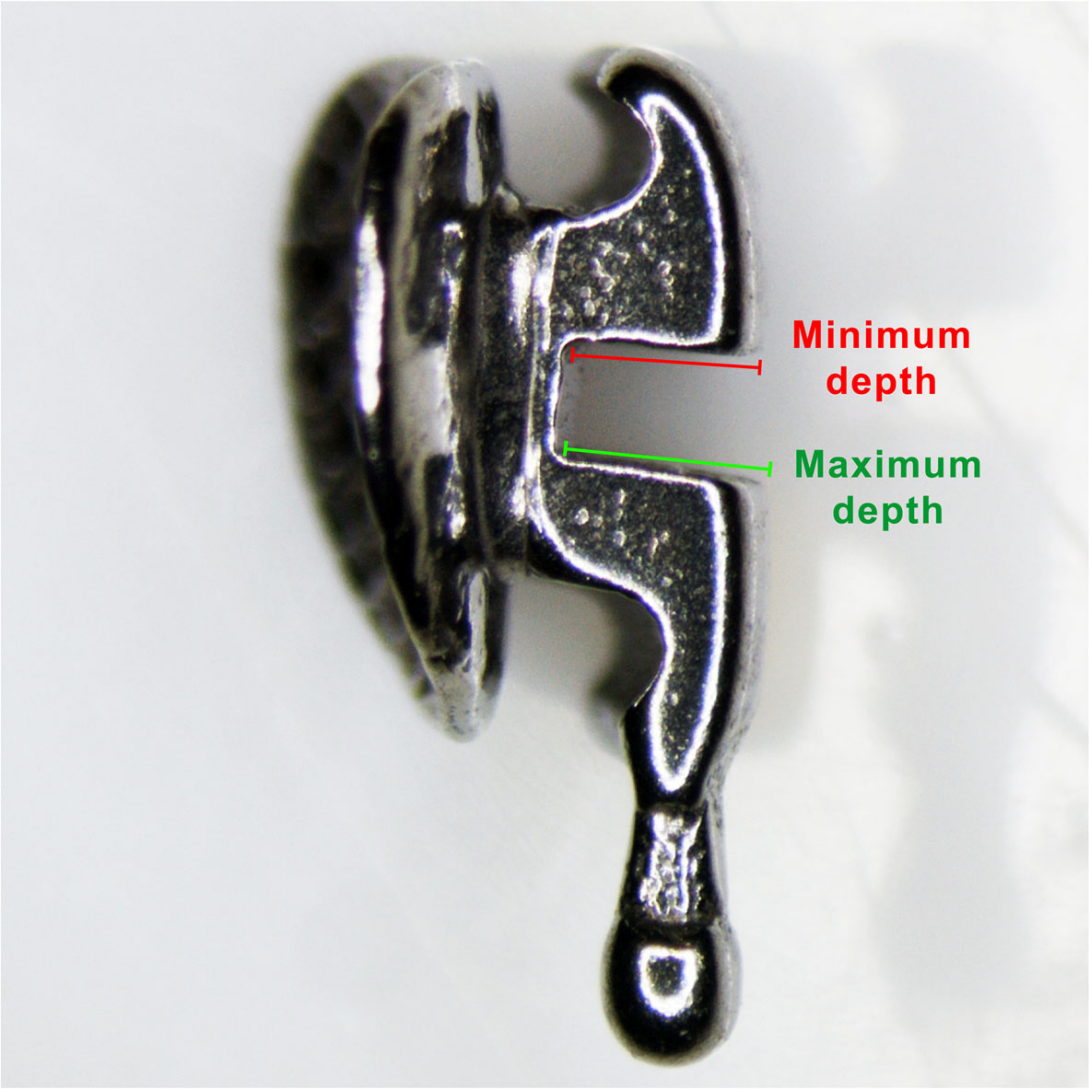
Definition of the slots’ maximum depth and minimum depth

The measurements of the bracket’s slot dimension were performed using a profile projector (V-12B Profile Projector, Nikon, Tokyo, Japan). Each bracket was placed with the distal side of the slot perfectly perpendicular to the observer (Fig. 2), and the slot height, the slot maximum depth, and the slot minimum depth were measured and recorded (Fig. 3).

### Archwire measurement

The archwires were cut into 4-cm straight segments and were placed in groups of five, homogeneous for size and manufacturer, into a cylindrical mould. One end of each segment was embedded into a hard, sticky wax (Ceracol; Zeta Industria Zingardi, Italy) in a perfect vertical position. This step was necessary to obtain a perfect orthogonal section of the wire during the subsequent procedures. After that, two-component epoxy resin (EpoxyCure 2™, Buehler, Lake Bluff, IL, USA) was poured into the cylindrical mould and cured according to the manufacturer’s instruction.

Each cylinder was sectioned into 5-mm-thick slices using a low speed diamond saw (Isomet® Low Speed Saw, Buehler, IL, USA) and then polished using a lapping machine (LS2, Remet S.a.s., Bologna, Italy) with abrasive papers (CarbiMet™, Buehler, IL, USA) at different grit size, which increased from 600 grit to 1200 grit.

The grinding phase was completed using specific polishing clots associated with progressive decreasing diamond suspension (MetaDi™ Monocrystalline Diamond Suspension, Buehler, IL, USA) of 9 μm, 3 μm, and 1 μm.

The prepared samples were preliminarily observed with a magnification of × 1 using an optical Greenough Stereo Microscope (S8 AP0, Leica Microsystems GmbH, Wetzlar, Germany), associated with a digital colour camera (EC3, Leica Microsystems GmbH, Wetzlar, Germany), to evaluate their morphologic aspects and the achievement of a satisfactory surface polishing.

Each sample was then observed with a field emission gun scanning electron microscope (SEM) under back-scattering electron (BSE) modality (GeminiSEM 500, Carl Zeiss Microscopy GmbH, Jena, Germany) at × 300 magnification. The BSE modality generates an image based on the atomic mean number of elements present in each specimen, and characterized by a black and white contrast where bright areas represent elements with a high mean atomic number, for example metals, while dark areas are relative to elements with a low atomic number, like epoxy resin.

The acquired images were analysed using an image processing programme (ImageJ v1.52K, National Institutes of Health, USA): each SEM image was first calibrated using the 100-μm-long ruler; then, the height, the width, and the curvature radius of all the four bevelled edges of each archwire section were measured by the same operator (GP) on a 1920 × 1080 pixels monitor. The height and the width were measured on a straight line connecting the most external points of each side. The edge bevel radius was measured through a multi-point selection and a fit-circle function.

### Torsional play calculation

**Fig. 4:**
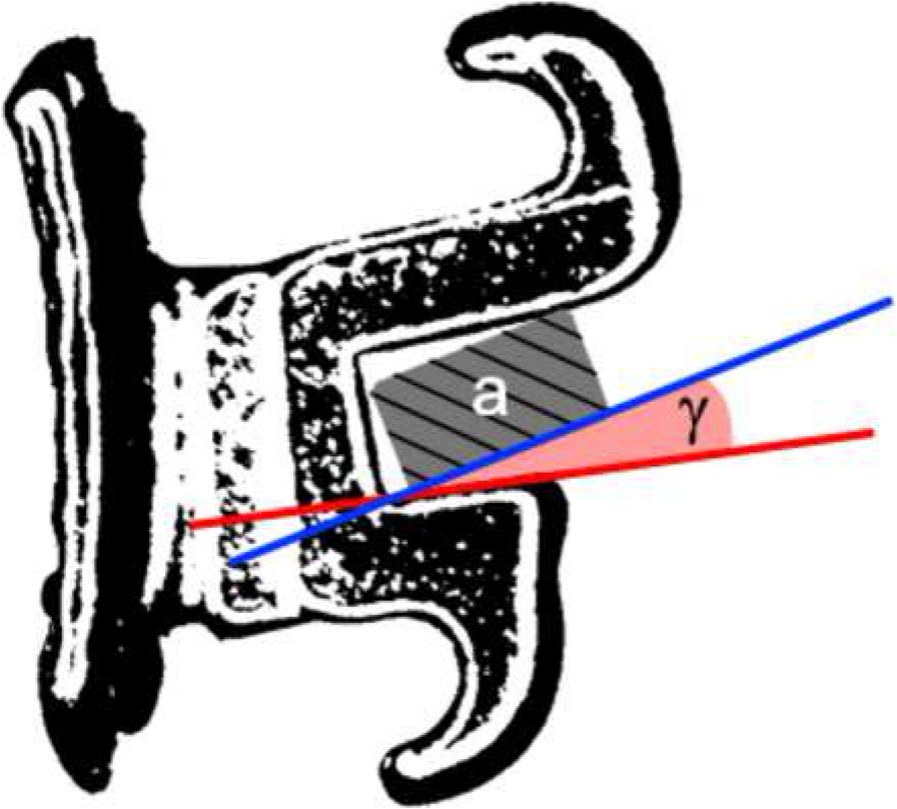
Depiction of the torsional play angle (γ) between the bracket’s slot and the archwire (a)

Knowing the bracket’s slot height (H), and the archwire’s height (h), width (w), bevel radius (r), and the distance between the centre of the archwire’s bevelled edges (d), it was possible to estimate with high accuracy the theoretical torsional play (γ) (Fig. 4) for each bracket/archwire system using the formula (1) proposed and validated by Meling et al. [9]:

$$ (1)\ \gamma =\arcsin \frac{H-2r}{d}-\arcsin \frac{h-2r}{d} $$

The torsional play of each one of the combination of brackets and archwires from the same manufacturer was calculated, and the resulting value was converted to arc degrees.

### Statistical analysis

To evaluate the error of the method, 25 brackets and 25 archwires were randomly selected using an online tool (www.randomizer.org) and measured twice at a 1-week interval. The random error between the two sets of measurements was calculated using the Dahlberg formula, while the presence of systematic errors was evaluated with Bland−Altman plots.

Descriptive statistics were calculated for all the variables.

A one-way ANOVA was used to evaluate the presence of significant differences between the slot dimensions of different brackets (UR1, UR3, UR4) within each group. A one-way ANOVA was also used to evaluate the presence of significant differences regarding archwire dimensions and torsional play between each group. For both tests, a Levene’s test was used to verify the assumption of data homoscedasticity. A Tukey’s HSD or a Games−Howell post hoc test was then calculated, depending on the homogeneity of variance testing.

After applying the Bonferroni correction, type I error was set at 0.004 for all tests.

Statistical analysis was done using SPSS software (IBM SPSS Statistics for Windows, Version 26.0., IBM Corp, Armonk, NY, USA).

## Results

Regarding the evaluation of the error of the method, the Dahlberg formula revealed a random error of between 0.65 ± 0.07 μm and 0.75 ± 0.09 μm (corresponding to 0.025 mil and 0.029 mil, respectively), while the Bland−Altman plots revealed no systematic errors.

The bracket’s slot measurements revealed that most of the specimina were generally oversized, with few exceptions (Table 2). Concerning slot height, all measurements were larger than 0.022 in., with Group 1 showing the largest variability (from + 4.1% to + 5.9%). The slot depth showed a greater variability, with Group 1 showing the greatest increment (from + 25.3% to + 50.3% for the maximum depth, and from +20.3% to +24.6% for the minimum depth), and Group 3 showing the greatest differences with the UR4 brackets having a smaller depth (− 12.1% and − 21.7% for the maximum and minimum depth, respectively). Within each group, there were statistically significant differences between slot measurements of different brackets for different teeth (Table 3).

**Table 2:** Descriptive statistics for bracket’s slot measurements divided by group and bracket type

	Group 1			Group 2			Group 3		
	UR1	UR3	UR4	UR1	UR3	UR4	UR1	UR3	UR4
Slot height	0.0233 ± 0.0006	0.0229 ± 0.0003	0.0232 ± 0.0003	0.0224 ± 0.0003	0.0223 ± 0.0004	0.0227 ± 0.0003	0.0222 ± 0.0003	0.0227 ± 0.0004	0.0223 ± 0.0004
Maximum slot depth	0.0421 ± 0.0007	0.0351 ± 0.0012	0.0376 ± 0.0012	0.0345 ± 0.0003	0.0300 ± 0.0001	0.0340 ± 0.0030	0.0299 ± 0.0010	0.0332 ± 0.0027	0.0246 ± 0.0002
Minimum slot depth	0.0343 ± 0.0010	0.0337 ± 0.0009	0.0349 ± 0.0008	0.0286 ± 0.0004	0.0279 ± 0.0003	0.0283 ± 0.0009	0.0253 ± 0.0011	0.0294 ± 0.0024	0.0219 ± 0.0014

Mean ± SD; values are expressed in inch

*Group 1* Astar Orthodontics, *Group 2* SIA, *Group 3* Sweden & Martina, *UR1* upper right central incisor bracket, *UR3* upper right canine bracket, *UR4* upper right first premolar bracket

**Table 3:** Results of one-way ANOVA, divided by each group, comparing slot measurements for different bracket types

	Levene statistics (*p* value)	*F* statistics	*p* value	UR1 vs UR3	UR1 vs UR4	UR3 vs UR4
	Group 1					
Slot height	0.153	3.20	0.054	^†^0.0004 (0.054)	^†^0.0001 (0.843)	^†^− 0.0003 (0.167)
Slot width (max)	0.334	140.01*	< 0.001	^†^0.007* (< 0.001)	^†^0.0045* (< 0.001)	^†^− 0.0025* (< 0.001)
Slot width (min)	0.774	4.98*	0.013	^†^0.0006 (0.241)	^†^0.0006 (0.299)	^†^− 0.0012* (0.009)
	Group 2					
Slot height	0.730	3.20	0.057	^†^0.0000 (0.923)	^†^− 0.0003 (0.138)	^†^− 0.0004 (0.065)
Slot width (max)	0.028	20.04*	< 0.001	^‡^0.0044* (< 0.001)	^‡^0.0004 (0.889)	^‡^− 0.0040* (0.005)
Slot width (min)	< 0.001	3.15	0.059	^‡^0.0007* (0.004)	^‡^0.0003 (0.662)	^‡^− 0.0004 (0.402)
	Group 3					
Slot height	0.290	6.47*	0.005	^†^− 0.0005* (0.007)	^†^− 0.0000 (0.892)	^†^0.0004* (0.021)
Slot width (max)	0.046	45.20*	< 0.001	^‡^− 0.0032* (0.012)	^‡^0.0053* (< 0.001)	^‡^0.0086* (< 0.001)
Slot width (min)	0.131	47.03*	< 0.001	^†^− 0.0041* (< 0.001)	^†^0.0034* (0.001)	^†^0.0075* (< 0.001)

*Group 1* Astar Orthodontics, *Group 2* SIA, *Group 3* Sweden & Martina, *UR1* upper right central incisor bracket, *UR3* upper right canine bracket, *UR4* upper right first premolar bracket

*Statistically significant for *p* < 0.05

^†^Mean difference in inch (*p* value) from Tukey’s HSD post hoc test

^‡^Mean difference in inch (*p* value) from Games-Howell post hoc test

**Fig. 5:**
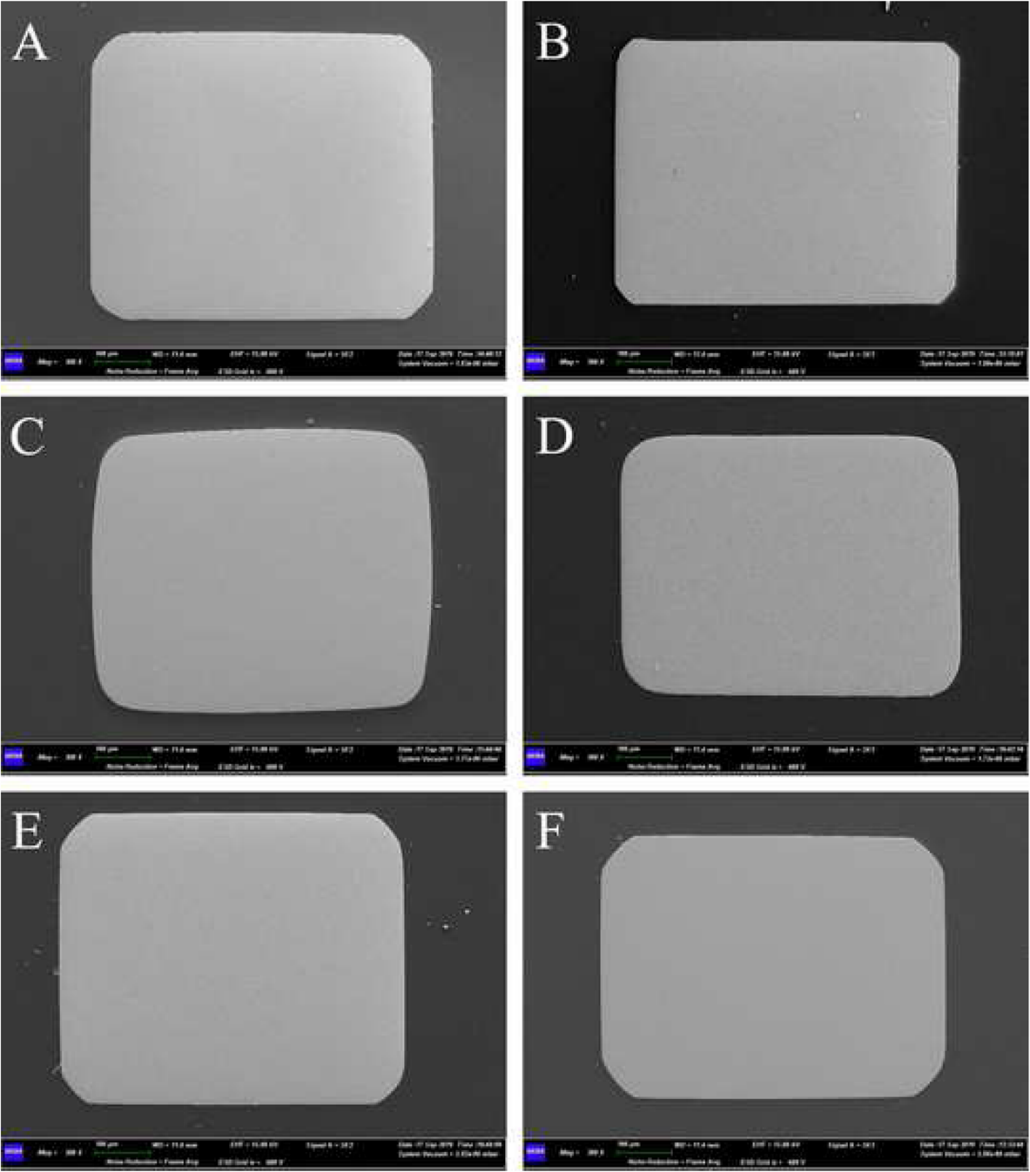
Images of archwires’ sections taken at 300× with a field emission gun scanning electron microscope under secondary electron modality. **a** 0.019 × 0.025″ stainless steel archwire from Group 1 (Astar Orthodontics Inc., Shanghai, China). **b** 0.021 × 0.025″ stainless steel archwire from Group 1. **c** 0.019 × 0.025″ stainless steel archwire from Group 2 (Sia Orthodontic Manufacturer S.r.l., Rocca D’Evandro, Caserta, Italy). **d** 0.021 × 0.025″ stainless steel archwire from Group 2. **e** 0.019 × 0.025″ stainless steel archwire from Group 3 (Sweden & Martina S.p.A, Due Carrare, Padova, Italy). **f** 0.021 × 0.025″ stainless steel archwire from Group 3

When looking at archwire measurements (Fig. 5), both height and width of all the tested archwires were generally undersized from − 0.4 to − 1.4%, except for the archwires from Group 3, which were slightly oversized from +0.4 to +0.8% (Table 4). All the measurements showed a statistically significant difference between the three groups (Table 5).

**Table 4:** Descriptive statistics for archwire’s measurements divided by group and size

	Group 1		Group 2		Group 3	
	0.019 × 0.025 archwire	0.021 × 0.025 archwire	0.019 × 0.025 archwire	0.021 × 0.025 archwire	0.019 × 0.025 archwire	0.021 × 0.025 archwire
Arch height	0.0190 ± 0.00009	0.0209 ± 0.00011	0.0189 ± 0.00009	0.0207 ± 0.00016	0.0191 ± 0.00004	0.0211 ± 0.00009
Arch width	0.0248 ± 0.00004	0.0249 ± 0.00010	0.0248 ± 0.00017	0.0248 ± 0.00015	0.0251 ± 0.00007	0.0252 ± 0.00015
Radius of edge bevel	0.0036 ± 0.00042	0.0039 ± 0.00048	0.0029 ± 0.00007	0.0030 ± 0.00010	0.0053 ± 0.00042	0.0049 ± 0.00064

Mean ± SD; values are expressed in inch

*Group 1* Astar Orthodontics, *Group 2* SIA, *Group 3* Sweden & Martina

**Table 5:** Results of one-way ANOVA, divided by each group, comparing slot measurements for different archwires

	Levene statistics (*p* value)	*F* statistics	*p* value	Group 1 vs Group 2	Group 1 vs Group 3	Group 2 vs Group 3
Height of 0.019 × 0.025 archwire	0.277	8.544*	0.005	^†^0.00009 (0.176)	^†^− 0.00011 (0.109)	^†^− 0.0002 (0.004)
Width of 0.019 × 0.025 archwire	0.016	16.320*	< 0.001	^‡^− 0.00004 (0.886)	^‡^− 0.00035* (< 0.001)	^‡^− 0.00031* (0.023)
Height of 0.021 × 0.025 archwire	0.281	14.535*	0.001	^†^0.00018 (0.098)	^†^− 0.00025* (0.023)	^†^− 0.00043* (< 0.001)
Width of 0.021 × 0.025 archwire	0.493	8.931*	0.004	^†^0.0001 (0.491)	^†^− 0.00025* (0.031)	^†^− 0.00036* (0.004)
Radius of edge bevel of 0.019 × 0.025 archwire	0.006	66.584*	< 0.001	^‡^0.00078* (0.029)	^‡^− 0.0017* (0.001)	^‡^− 0.00248* (< 0.001)
Radius of edge bevel of 0.021 × 0.025 archwire	0.006	21.184*	< 0.001	^‡^0.00094* (0.023)	^‡^− 0.00098 (0.065)	^‡^− 0.00192* (0.005)

*Group 1* Astar Orthodontics, *Group 2* SIA, *Group 3* Sweden & Martina

*Statistically significant for *p* < 0.05

^†^Mean difference in inch (*p* value) from Tukey’s HSD post hoc test

^‡^Mean difference in inch (*p* value) from Games-Howell post hoc test

About the measurement of the curvature of the bevelled edges, the values reported in Table 4 are a mean of the four edges of each archwire type. There was a great variability of the edge bevel’s curvature between different manufactures, with archwires from Group 3 showing rounder edges and Group 2 showing the most squared edges (Table 4). Those differences were statistically significant (Table 5).

Considering the torsional play calculations, the use of a 0.019 × 0.025″ archwire resulted in a torsional play ranging from 11° (Group 2) to nearly 16° (Group 1 and 3), while the use of a 0.021 × 0.025″ archwire resulted in smaller values between 4 and 8.6° (Table 6).

**Table 6:** Descriptive statistics for torsional play measurements divided by group and size

	Group 1		Group 2		Group 3	
	0.019 × 0.025 archwire	0.021 × 0.025 archwire	0.019 × 0.025 archwire	0.021 × 0.025 archwire	0.019 × 0.025 archwire	0.021 × 0.025 archwire
Torsional play for UR1 bracket	15.84 ± 0.68 (15.00–16.51)	8.60 ± 0.54 (8.05–9.50)	11.42 ± 0.29 (11.05–11.74)	5.32 ± 0.52 (4.64–5.85)	13.44 ± 0.89 (12.43–14.31)	4.07 ± 0.44 (3.52–4.59)
Torsional play for UR3 bracket	14.19 ± 0.60 (13.47–14.80)	7.08 ± 0.48 (6.62–7.86)	11.07 ± 0.28 (10.70–11.38)	5.00 ± 0.52 (4.31–5.51)	15.86 ± 1.05 (14.68–16.88)	6.08 ± 0.57 (5.46–6.80)
Torsional play for UR4 bracket	15.42 ± 0.66 (14.61–16.08)	8.22 ± 0.52 (7.69–9.08)	12.50 ± 0.29 (12.12–12.81)	6.32 ± 0.53 (5.63–6.83)	13.91 ± 0.92 (12.87–14.82)	4.47 ± 0.46 (3.90–5.03)

Mean ± SD (range); values are expressed in arc degrees

*Group 1* Astar Orthodontics, *Group 2* SIA, *Group 3* Sweden & Martina, *UR1* upper right central incisor bracket, *UR3* upper right canine bracket, *UR4* upper right first premolar bracket

There were statistically significant differences between different groups for every combination of bracket type and archwire dimension (Table 7).

**Table 7:** Results of one-way ANOVA comparing the values of torsional play between different groups

	Levene statistics (*p* value)	*F* statistics	*p* value	Group 1 vs Group 2	Group 1 vs Group 3	Group 2 vs Group 3
Torsional play for a 0.019 × 0.025 archwire in a UR1 bracket	0.008	55.271 *	< 0.001	^‡^4.42* (< 0.001)	^‡^2.41* (0.004)	^‡^− 2.01* (0.012)
Torsional play for a 0.019 × 0.025 archwire in a UR3 bracket	0.003	57.566 *	< 0.001	^‡^3.12* (< 0.001)	^‡^− 1.67* (0.046)	^‡^− 4.79* (0.001)
Torsional play for a 0.019 × 0.025 archwire in a UR4 bracket	0.007	23.613 *	< 0.001	^‡^2.92* (< 0.001)	^‡^1.51* (0.045)	^‡^− 1.41 (0.051)
Torsional play for a 0.021 × 0.025 archwire in a UR1 bracket	0.892	108.144*	< 0.001	^†^3.27* (< 0.001)	^†^4.53* (< 0.001)	^†^1.26* (0.005)
Torsional play for a 0.021 × 0.025 archwire in a UR3 bracket	0.754	20.019*	< 0.001	^†^2.09* (< 0.001)	^†^1.01* (0.025)	^†^− 1.08* (0.017)
Torsional play for a 0.021 × 0.025 archwire in a UR4 bracket	0.871	68.683*	< 0.001	^†^1.90* (< 0.001)	^†^3.75* (< 0.001)	^†^1.85* (< 0.001)

*Group 1* Astar Orthodontics, *Group 2* SIA, *Group 3* Sweden & Martina, *UR1* upper right central incisor bracket, *UR3* upper right canine bracket, *UR4* upper right first premolar bracket

*Statistically significant for *p* < 0.05

^†^Mean difference in arc degrees (*p* value) from Tukey’s HSD post hoc test

^‡^Mean difference in arc degrees (*p* value) from Games-Howell post hoc test

## Discussion

Straight-wire techniques require a strict contact between archwires and the brackets’ slot to express the movement’s prescription, in particular regarding third-order information. Ideally, this would be achieved by an archwire that fills the slot completely, but such an archwire will be difficult to engage [1]. In accordance with the results of the present study, enlarging the slot and decreasing the wire’s cross section are some common manufacturer’s dispositions to simplify the insertion of an archwire [2, 14], which are acceptable as long as they do not interfere with a complete torque expression.

Only a few studies [1, 6, 15] have attempted to evaluate dimensional discrepancies from standards and to quantify the real influence on torque expression, but often overlooking some parameters.

As stated above, the findings of the present study confirmed what was found in the literature, namely that slot height measurements were always above the nominal value (0.022 in.). In addition, Group 3 showed statistically significant differences in slot height between the UR1, UR3, and UR4 bracket, with the UR3 bracket being more oversized than the other two.

There was a great variability regarding both maximum depth and minimum depth between different manufacturers and within groups for different bracket types; although this parameter has a limited impact on torsional play, it is an expression of the large variability of slot morphology across different brackets and manufacturers.

The three bracket systems studied were produced through different industrial processes (milling, MIM, MIM bracket with a milled slot) that have some advantages but also some defects; for example, MIM is economic and good for complex morphology [16] but is characterized by surface porosity and consequently less mechanical strength [17]. Milling is the most precise system for simple morphology but more expensive and time-consuming [18] but it is possible to take relative advantage from MIM and milling, using them in combination [16]. However, in the present study, this was not always respected, since the brackets made entirely from milling were not the most precise ones (Table 2). Nevertheless, many other factors related to the construction process can have an influence on the bracket’s final dimensions; therefore, any conclusion regarding the industrial process used is beyond the scope of the present study and cannot be drawn.

Archwire dimensions showed a different variability, with Groups 1 and 2 having in general an undersized cross section especially regarding height, while Group 3 had an oversized section. This trend was similar for 0.019 × 0.025″ and 0.021 × 0.025″ archwires. On the other hand, the most important aspect seems to be the radius of the edge bevel, as demonstrated by Meling et al. [8] who found that the theoretical estimation of torsional play was far more accurate and closer to the real value when taking into account the edge bevel. The effect of the edge bevel is also linked to the archwire’s material [6], and its influence on the expression of third-order information is also related to a reduction of the cross-sectional area that makes the wire less stiff [3, 19, 20].

The archwires from the three groups presented very different bevels, and this is justified by the absence of ISO norms regarding this aspect of the archwire’s properties. Rectangular archwires result from a rolling process with a Turks head that is necessarily accompanied by a certain amount of wire rounding, which results in an edge bevel that represents a critical factor for torque expression [5] but is also useful to facilitate wire engagement [2] and to avoid cuts or damages to the patient’s soft tissues [21].

The torsional play measurements showed statistically significant differences among all the groups for every combination of bracket type and archwire section (Table 7).

Overall, considering a 0.019 × 0.025″ stainless steel archwire, which is the working archwire for many orthodontic techniques, the torsional play can lead to a loss of torque information of from 10.7 to 16.9°. Considering a theoretical play of 10.5° for a 0.019 × 0.025″ wire in a 0.022 × 0.028″ slot [5], the present data suggest that the dimensional variability due to manufacturing can lead up to an additional 61% increase of torsional play. Within each bracket/archwire system from the same manufacturer, the torsional play was different between the UR1, UR3, and UR4 brackets, meaning that in a single bonded jaw there could be a loss of torque information varying for each tooth from 1.43 to 2.42°. Large differences were present also between the three groups: Group 2 showed the smallest torsional play values, and Group 1 showed the largest torsional play values for UR1 and UR4 brackets, while the largest torsional play value for UR3 was measured in Group 3. These results can be considered also clinically significant, since the smallest difference observed, which is of about 2° for the upper central incisor, of 1.7° for the upper canine, and of 1.4° for the upper first premolar, represents respectively the 12%, the 24%, and the 20% of the standard MBT prescription for each bracket. Examining all the dimensional characteristics that led to this outcome, the edge bevel’s radius probably emerges as the most important one. Looking, for example, at the comparison between Group 2 and Group 3 and using the UR1 bracket as a reference, the slot height was larger in Group 2 than in Group 3, and the 0.019 × 0.025″ archwire from Group 2 had the smallest height of all the three groups, while the same archwire from Group 3 was even oversized. One would infer from this data that torsional play will be higher in Group 2, but in reality the archwires from Group 3 had a rounder edge bevel that produced a torsional play 2° greater than Group 2, which on the contrary had the smallest edge bevel’s radius (Tables 5 and 7). Manufacturer 3 produced an oversized archwire to fill the slot better and tried to facilitate the insertion of such an archwire by greatly rounding its edges, but, in the end, this led to a worst loss of third-order information.

The same considerations can be made regarding the 0.021 × 0.025″ stainless steel archwires. This archwire is less used than the 0.019 × 0.025″, but offers a significant advantage in terms of expression of third-order information, filling the slot and showing a smaller torsional play ranging from 4.07 to 8.6°, almost half of the values observed with the 0.019 × 0.025″ archwire. Considering a theoretical play of 2.3° for a 0.021 × 0.025″ wire in a 0.022 × 0.028″ slot [5], the present data suggest that the dimensional variability due to manufacturing can result in an increase of torsional play up to three times the original value. Also with the 0.021 × 0.025″ archwire, the torsional play was different among the UR1, UR3, and UR4 brackets within each bracket/archwire system from the same manufacturer. This means that even when filling the slot with a stiffer wire, in a single bonded jaw, there could be a loss of torque information from one tooth to another of about 1.32–2.01°. In addition, statistically significant differences in torsional play were present among different manufacturers, ranging from 1.01 to 4.53°: Group 1 showed the greatest torsional play values, and Group 3 presented the smallest values for UR1 and UR4 brackets, while Group 2 showed the smallest values for UR3 bracket. These results are slightly different from those reported for the 0.019 × 0.025″ archwires, because in Group 3 the edge bevel’s radius of the 0.021 × 0.025″ archwire was smaller than the 0.019 × 0.025″ archwire, while on the other hand in Group 2 the edge bevel’s radius was larger in the 0.021 × 0.025″ archwire than in the 0.019 × 0.025″ archwire (Table 4). The differences in torsional play observed between the three manufacturers were clinically significant, ranging from 15 to 27% of the bracket’s prescription (Table 7).

The results of the present study are in substantial agreement with those of other authors. Joch et al. [1] evaluated the theoretical torsional play in a 0.022 × 0.028″ slot without considering the edge bevels and found values ranging between 4.5 and 11.3° for 0.020 × 0.025″ archwires, and between 5.9 and 11.7° for 0.019 × 0.025″ archwires. Other studies that also considered the effect of the edge bevel found higher torsional play values, ranging from 7.8 to 23° for different archwire sizes in a 0.018″ bracket [15]. Similarly, Lombardo et al. [6], considering an ideal slot height of 0.018″ or 0.022″ and real archwire’s measurements taken with a digital gauge, found that torsional play values ranged from 3.28 to 34.17°.

The observed torsional play values are sometimes able to nullify most torque prescription of common pre-adjusted appliances. Considering a multibracket appliance with MBT prescription, for example, the UR1 bracket should deliver to the tooth a nominal palatal root torque of 17° [22], but the torsional play of a 0.021 × 0.025″ archwire can lead to a torque loss of from 24.0% (Group 3) to 50.6% (Group 1), thus achieving a real torque of only 12.93° or 8.39°. Considering the worst torsional play value observed for a UR1 brackets and a 0.021 × 0.025″ archwire among all the three manufacturers, torque loss can reach 55.9% and real torque can be of only 7.5°. If we consider the UR3 and the UR4 brackets, which have a − 7° prescription of buccal root torque, the measured torsional play (Table 6) has an even larger clinical impact.

In light of these findings, the claims of some manufacturers about the advantages of new prescriptions that sometimes differ by few degrees from other ones become meaningless.

Torque expression also depends on other factors, like bracket positioning, archwire material properties, and wire ligation. In particular, stainless steel ligatures assure a better slot/archwire engagement and a better torque control than elastomeric ligatures, which deteriorate rapidly in the oral environment [2, 23].

Certainly, the limitations presented by the inherent slot/archwire torsional play can be overcome by the clinician through wire bends, auxiliaries, or special ligations. However, it is important for the clinician to know the real possibilities of the bracket/archwire system that he or she is using: this allows a better understanding of the prescription being used and an awareness and timely using of auxiliary techniques that are necessary to achieve the desired tooth movements, regardless of the inherent torsional play that is always present in every appliance.

Regarding the limitations of the present study, it was not possible to incorporate an inter-lot variation for some manufacturers due to technical reasons. In addition, the studied archwires showed a shape variability that is not accounted for by the formula (1) used; therefore, the measurements of the real torsional play values will be the next step of this investigation.

## Conclusions

- Bracket slot’s heights are constantly oversized, but some producers are more adherent to nominal values.

- Archwires are usually slightly undersized, but oversized archwires were also observed.

- Edge bevels are unavoidable consequences of industrial production of rectangular archwires and are extremely variable from one product to another, but they have a great impact on torsional play and the expression of third-order information. A more detailed description of this characteristic from the manufacturers, and the definition of tighter standards, would be advisable.

- The torsional play is usually significant and can nullify most of the common used prescriptions.

## Data Availability

The datasets used and/or analysed during the current study are available from the corresponding author on reasonable request.

## Abbreviations

MBT: McLaughlin-Bennett-Trevisi
ISO: International Organization for Standardization
MIM: Metal injection moulding
CNC: Computer numerical control
SEM: Scanning electron microscope
BSE: Back-scattering electron
UR1: Bracket for the upper right central incisor
UR3: Bracket for the upper right canine
UR4: Bracket for the upper right first premolar
